# Function of NEK2 in clear cell renal cell carcinoma and its effect on the tumor microenvironment

**DOI:** 10.1097/MD.0000000000037939

**Published:** 2024-05-17

**Authors:** Peng Tang, Gangfu Zheng, Congcong Xu, Nengfeng Yu, Jiaqi Du, Liqian Hu, Zhan Zhou, Yichun Zheng

**Affiliations:** a The Fourth Affiliated Hospital, Zhejiang University School of Medicine, Yiwu, China; b Department of Urology, The First People’s Hospital of Linping District of Hangzhou, Hangzhou, China; c The Second Affiliated Hospital, Zhejiang University School of Medicine, Hangzhou, China; d Innovation Institute for Artificial Intelligence in Medicine and Zhejiang Provincial Key Laboratory of An-ti-Cancer Drug Research, College of Pharmaceutical Sciences, Zhejiang University, Hangzhou, China.

**Keywords:** clear cell renal carcinoma, immune cell infiltration, NEK2, prognosis

## Abstract

**Background:**

Previous studies have revealed the critical functions of NEK2 in controlling the cell cycle which is linked to poor prognosis in multiple tumor types, but less research has been devoted to clear cell renal cell carcinoma (ccRCC).

**Methods:**

We downloaded clinical data from the gene expression omnibus (GEO) and TCGA databases together with transcriptional and mutational datasets. Strongly coexpressed genes with NEK2 were extracted from TCGA-KIRC cohort, and were submitted to Gene Ontology (GO) and Kyoto Encyclopedia of Genes and Genomes (KEGG) for functional analyses. According to NEK2 levels, the survival status, mutational characteristics, response to immunotherapy and sensitivity to drugs of the patients were studied. The potential correlations between NEK2 levels and immune cell state as well as immune cell infiltration were examined using the GEPIA, TIMER and TISIDB databases. Double immunofluorescence (IF) was performed to identify the NEK2 overexpression and relationship with CD8 in ccRCC.

**Results:**

The NEK2 gene was overexpressed and would enhance the nuclear division and cell cycle activities in ccRCC. ccRCC patients with high NEK2 expression had worse clinical outcomes, higher mutation burden and better therapeutic response. Moreover, NEK2 gene overexpression was positively related to various immune cell marker sets, which was also proved by validation cohort, and more infiltration of various immune cells.

**Conclusion:**

ccRCC patients with NEK2 high expression have a poorer prognosis than those with NEK2 low expression, resulting from its function of promoting proliferation, accompanied by increased infiltration of CD8 + T cells and Tregs and T-cell exhaustion and will respond better to proper treatments.

## 1. Introduction

Renal cell carcinoma (RCC) is one of the most prevalent malignancies in the world. Statistics show that 179,368 patients died from RCC-related causes and that 431,288 new cases of RCC were diagnosed globally in 2020.^[1]^ The most typical histological subtype of RCC and the leading cause of death from RCC is clear cell renal cell carcinoma (ccRCC).^[2,3]^ RCC was perhaps the exemplar of malignancies characterized by metabolic reprogramming,^[4–8]^ which refers to glucose metabolism,^[9–11]^ the tricarboxylic acid cycle,^[12,13]^ lipid metabolism^[14,15]^ and amino acid metabolism.^[16,17]^ Therefore kidney cancer is referred to as a metabolic disease. Although primary localized ccRCC can be treated with surgical resection, the treatment of advanced ccRCC remains a clinical challenge as a result of having low radiation and susceptibility to adjuvant chemotherapy.^[18,19]^ Immune checkpoint inhibitors (ICIs) have recently been shown to be beneficial in treating patients with metastatic renal cancer.,^[20,21]^ but death and morbidity rates are still high.^[22]^ At present, there is a lack of biomarkers to determine the prognosis of ccRCC patients. Therefore, identifying biomarkers of ccRCC for early diagnosis and evaluation of prognosis is vital for the management and treatment of ccRCC patients.

## 2. Materials and methods

### 2.1. Data resources

The Gene Expression Omnibus (GEO) database was used to summarize the transcriptional patterns of 2 independent cohorts that included paired non-tumor tissues and tumor tissues. Data on clinicopathological characteristics and TCGA-KIRC mRNA expression profiles were collected from the TCGA database (https://cancergenome.nih.gov/). A total of 607 samples—535 original tumor samples and 72 normal samples—were included in the TCGA-KIRC project.

### 2.2. Validation cohort

To validate the bioinformatic results, we collected 16 cases of ccRCC who were confirmed with ccRCC and underwent partial or radical nephrectomy in the Fourth Affiliated Hospital Zhejiang University School of Medicine. We obtained corresponding non-tumor normal tissues from 8 high-stage tumor samples and tumor tissues from all 16 samples. Tissues were then paraffin-embedded, sectioned, and mounted onto slides. This study was approved by the Ethical Committees of the Fourth Affiliated Hospital Zhejiang University School of Medicine, and all procedures were strictly executed by the principles of the Declaration of Helsinki, and written informed consent was obtained from each subject.

### 2.3. Analysis of NEK2 expression

On the UALCAN website,^[23,24]^ the expression of NEK2 in various malignancies was investigated. To verify whether there was statistical significance between tumor samples and normal samples, GEPIA^[25]^ was employed. In both UALCAN and the GEPIA website, we verified the strong association between NEK2 expression and the clinicopathological characteristics of ccRCC patients.

### 2.4. NEK2 coexpression analysis

The “psych” package in the R platform was used to identify NEK2 coexpressed genes. TIMER was used to confirm the correlation between NEK2 and genes of interest. Using GeneMANIA, we also built PPI networks based on these targets. The TCGA-KIRC cohort was used for all analyses. Cor > 0.4 and *P* < .05 were viewed as the benchmarks for screening strong positive coexpressed genes.

### 2.5. Survival analysis

To explore the survival possibility of ccRCC samples with different NEK2 expression levels, we used the “survival” package on the R studio platform. The graphics were formed through the “survminer” package, which contained the value of log-rank *P* values.

### 2.6. Gene set enrichment analysis

To investigate the underlying pathway of the strong positive coexpressed genes, those genes were analyzed through the “clusterProfiler” package for GO functional and KEGG pathway analyses. Additionally, we employed the “GSVA” package gene set enrichment analysis (GSEA). Specific pathways’ normalized enrichment scores were assessed. Each analysis used gene set permutations 1000 times, and a *P* value of .05 or lower was regarded as statistically significant. The functions “ggplot” and “gseaplot2” from the “enrichplot” and “ggplot2” packages, respectively, were used to create the plots.

### 2.7. Somatic mutation analysis

The TCGA-GDC database contained mutation information for 335 ccRCC patients. Based on the median transcriptional levels of the NEK2 gene in the original cohort, we divided the mutation queue into 2 groups (NEK2high and NEK2low). The 2 groups’ mutation pictures were drawn using the “oncoplot” function in the “maftools” package of the “R studio” platform, and the mutant genes with noticeably different distributions were assessed using the “mafCompare” function in the “maftools” package.^[26]^ Subsequently, we explored the tumor mutational burden (TMB) of ccRCC patients between the 2 groups.

### 2.8. Immune infiltrate analysis

We submitted NEK2 gene to the TIMER database (http://timer.comp-genomics.org/), a website for the analysis of immune infiltrates in human malignancies,^[19]^ to assess the role of NEK2 in immune infiltrates in ccRCC. To study the relationship between NEK2 expression and immune-related genes (IRGs), KIRC data downloaded from TCGA was analyzed using TISIDB (http://cis.hku.hk/TISIDB), a website dedicated to studying the relationship between tumors and the immune system.^[27]^ In this study, we investigated the relationship between NEK2 and immune biomarker expression and immune cell infiltration in the TCGA-KIRC project using Spearman correlation analysis.

### 2.9. Evaluation of therapeutic efficacy

We employed computational techniques to evaluate the therapeutic sensitivity of immunotherapy and chemotherapy between the 2 risk groups. The Cancer Immunome Atlas (TCIA; https://tcia.at/), as previously published,^[28]^ was used to predict the immunotherapeutic responses (anti-PD-1 and anti-CTLA4) of the 2 groups based on the gene expression levels. For targeted therapeutic drug analysis, we adopted the “pRRophetic” package, which evaluates the half-maximal inhibitory con-centration through ridge regression based on the Genomics of Drug Sensitivity in Cancer (GDSC; https://www.cancerrxgene.org/) database.^[29]^ Additionally, using data from the DrugBank (www.drugbank.ca) database, we also examined the expression of the target genes of various medicines.

### 2.10. Double immunofluorescence (IF)

Deparaffinized patient ccRCC tissue slices were then treated with EDTA buffer (pH 8.0) at 95 to 100°C for 15 minutes. The slides were blocked with 3% bovine serum albumin in PBS for 30 minutes after being washed 3 times with PBS. The slides were treated with a mixture of anti-NEK2 antibody (1:50, 24171-1-AP) and an-ti-CD8 antibody (1:100, ab251596, Abcam) overnight at 4°C. The slides were then washed, treated with a combination of 2 secondary antibodies for 50 minutes at room temperature, and then stained with DAPI. A confocal laser scanning microscope (Nikon Eclipse Ti) was employed to photograph the cells after 3 PBS washes.

### 2.11. Statistical analysis

The chi-squared test was used to determine the connection between NEK2 levels and clinicopathological features. Throughout the investigation, SPSS Inc. (Chicago, IL) version 23.0 statistical package was utilized. Statistics were considered significant at *P* < .05.

## 3. Results

### 3.1. High expression of NEK2 is associated with tumor progression and unfavorable prognosis in patients with ccRCC

**Figure 1.**
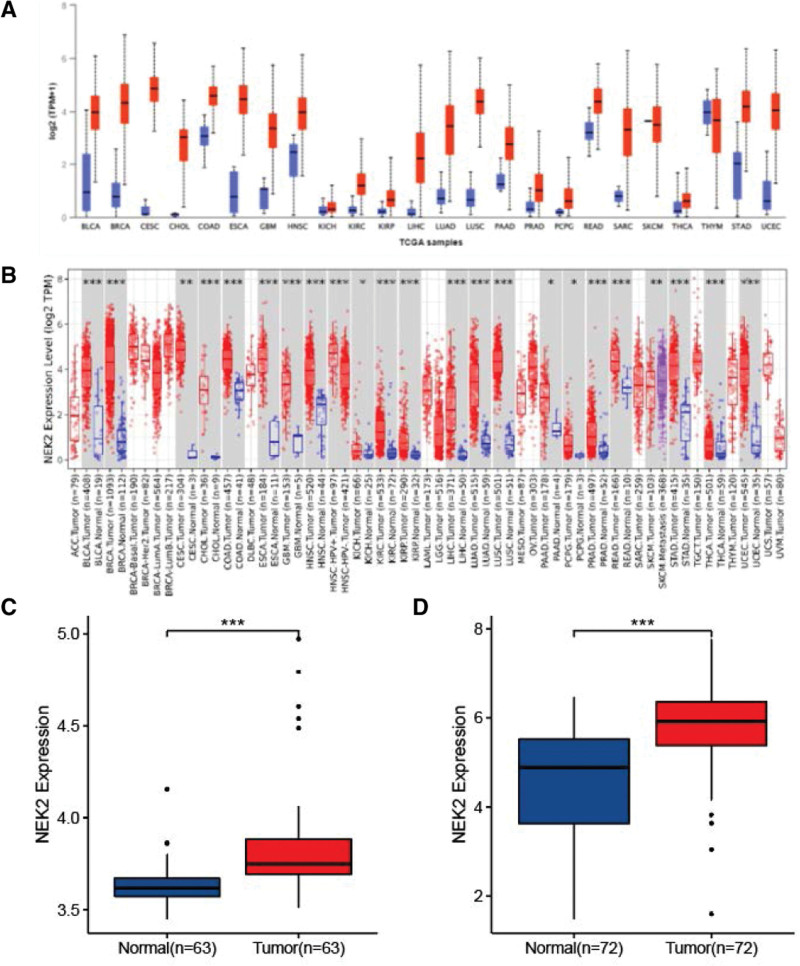
Variable forms of human malignancies express different amounts of NEK2 (*P < .05, **P < .01, ***P < .001). (A–B) UALCAN and TIMER were used to compare NEK2 expression levels between various tumor types from The Cancer Genome Atlas (TCGA) database. (C–D) The NEK2 expression difference between tumor and non-tumor tissues of ccRCC patients based on the GEO database. ccRCC = clear cell renal cell carcinoma, GEO = gene expression omnibus.

To gain a clear understanding of the impact of NEK2 in 24 tumor types, we employed UALCAN pan-cancer view feature. As shown in Figure 1A, NEK2 is dysregulated in diverse tumor types and upregulated mostly, except pheochromocytoma and paraganglioma, skin cutaneous melanoma and thymoma. The outcomes of the TIMER database, which incorporates RNA-seq data of tumors retrieved from TCGA, are comparable (Fig. 1B). It demonstrated that NEK2 is crucial to the emergence and metastasis of most tumor. Next, we studied NEK2 expression in ccRCC using the UALCAN and GEPIA databases (Supplementary Figure 1, http://links.lww.com/MD/M255). The outcomes demonstrated that NEK2 gene expression was higher in KIRC tumor tissues (T) than in non-tumor tissues (N). GSE46699 and GSE53757 also supported the overexpression phenotype of NEK2 (Fig. 1C–D).

**Figure 2.**
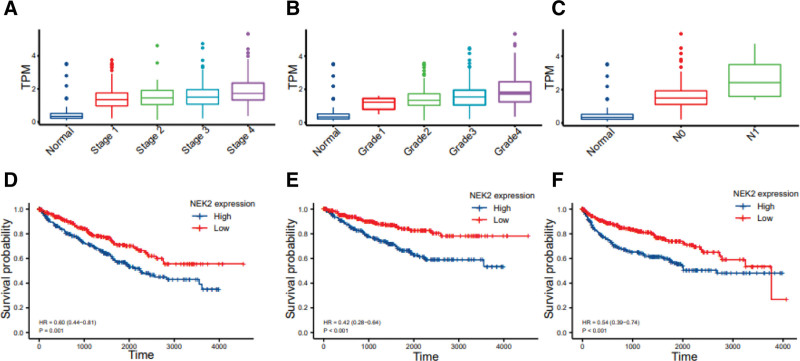
NEK2 expression levels along with clinicopathological characteristics in ccRCC. (A–C) Correlation between NEK2 expression level and clinicopathological parameters (cancer stage, cancer grade and lymph node stage) of ccRCC in the TCGA-KIRC cohort. (D–F) Survival curves of OS, DSS and PFI in ccRCC patients from TCGA database. ccRCC = clear cell renal cell carcinoma, DSS = disease-specific survival, PFI = progression-free interval, TCGA = The Cancer Genome Atlas.

Then, we examined NEK2 expression along with clinicopathological characteristics in the TCGA-KIRC cohort to ascertain the effects of different NEK2 expression levels on ccRCC patients. As shown in Figure 2A, the NEK2 gene significantly correlated positively with the stage of the tumor, (Fig. 2B). It showed NEK2 expression was positively correlated with tumor stage. In our own validation cohort, double immuno-fluorescence also indicated greater expression of the NEK2 gene in advanced tumor stages (Fig. 7F). Moreover, the expression of NEK2 in different tumor grades was also significantly different (Fig. 2B). Taking nodal and tumor metastasis status into consideration, the results implied that the higher the level of NEK2 expression was, the worse the nodal and tumor metastasis status was (Fig. 2C, Supplementary Figure 2, http://links.lww.com/MD/M256). Between any 2 groups, there was statistical significance. The above results all could be repeated in the GEPIA and UALCAN database (Supplementary Figure 3, http://links.lww.com/MD/M257). Then, we explored the influences in the TCGA cohort in-depth. Patients were divided evenly into 2 groups (NEK2high and NEK2low) by cutting off the median expression of NEK2 levels. The results revealed a strong correlation between the overexpression of NEK2 and the T stage, N stage, M stage, pathologic stage, and pathologic grade (Table 1, all *P* < .05), suggesting that the NEK2 gene may be essential for the development of ccRCC. In addition, NEK2 expression was statistically correlated with clinical survival outcomes in the chi-square analysis (Table 1). Therefore, we performed survival analysis in the TCGA cohort and found that NEK2 overexpression affects ccRCC patients’ poor overall survival (OS), disease-specific survival and progression-free interval (Fig. 2D–F). After univariate analysis (Table 2), multivariable Cox regression demonstrated that after adjusting for the influence of age, the overexpression of NEK2 was one of the independent and important prognostic variables for OS in patients with ccRCC (HR = 1.29, *P* < .001) (Supplementary Figure 4, http://links.lww.com/MD/M258).

**Table 1:** Clinicopathological characteristics related to NEK2 expression status in the TCGA-KIRC project.

Characteristics	TCGA cohort (N = 535)	NEK2 expression		χ^2^	*P* value
		High N = 267 (%)	Low N = 268 (%)		
Age					
≥70 yr		64 (47.1)	72 (52.9)	0.413	.521
<70 yr		198 (50.3)	196 (49.7)		
NA		5 (100)	0		
Gender					
Male		187 (53.6)	162 (46.4)	5.423	.02
Female		80 (43.0)	106 (57.0)		
T					
T1-T2		147 (42.6)	198 (57.4)	20.695	<.001
T3-T4		120 (63.2)	70 (36.8)		
N					
N0		125 (52.1)	115 (47.9)	8.895	.003
N1		15 (93.75)	1 (6.25)		
NX		127 (45.5)	152 (54.5)		
M					
M0		205 (48.3)	219 (51.7)	11.502	.001
M1		54 (69.2)	24 (30.8)		
MX		8 (24.2)	25 (75.8)		
Pathologic stage					
Stages I–II		138 (42.2)	189 (57.8)	18.701	<.001
Stages III–IV		126 (61.5)	79 (38.5)		
NA		3 (100)			
Pathologic grade					
Grade I–II		96 (39.2)	149 (60.8)	256.112	<.001
Grade III–IV		169 (59.9)	113 (40.1)		
NA		2 (25.0)	6 (75.0)		
Status					
Alive		158 (43.9)	202 (56.1)	17.218	<.001
Dead		108 (63.2)	63 (36.8)		
NA		1 (25)	3 (75)		

Statistical significance was determined by the chi-square test or Fisher exact test.

TCGA = The Cancer Genome Atlas.

**Table 2:** Univariate Cox logistic regression analysis of OS in the TCGA cohorts.

Covariates	Univariate analysis		*P* value
	HR	95%CI	
Age	0.03	1 (1–1)	6.60E-06
Gender	0.068	1.1 (0.78–1.5)	.67
Grade	0.84	2.3 (1.9–2.8)	7.60E-16
Stage	0.65	1.9 (1.7–2.2)	6.70E-22
Tstage	0.67	2 (1.7–2.3)	1.40E-15
Nstage	1.2	3.4 (1.8–6.4)	.00017
Mstage	1.5	4.4 (3.2–6)	1.90E-20
NEK2.expression	0.53	1.7 (1.2–2.3)	.00079

*P* values with statistical significance (*P* < .05) are marked bold.

OS = overall survival, TCGA = The Cancer Genome Atlas.

**Figure 3.**
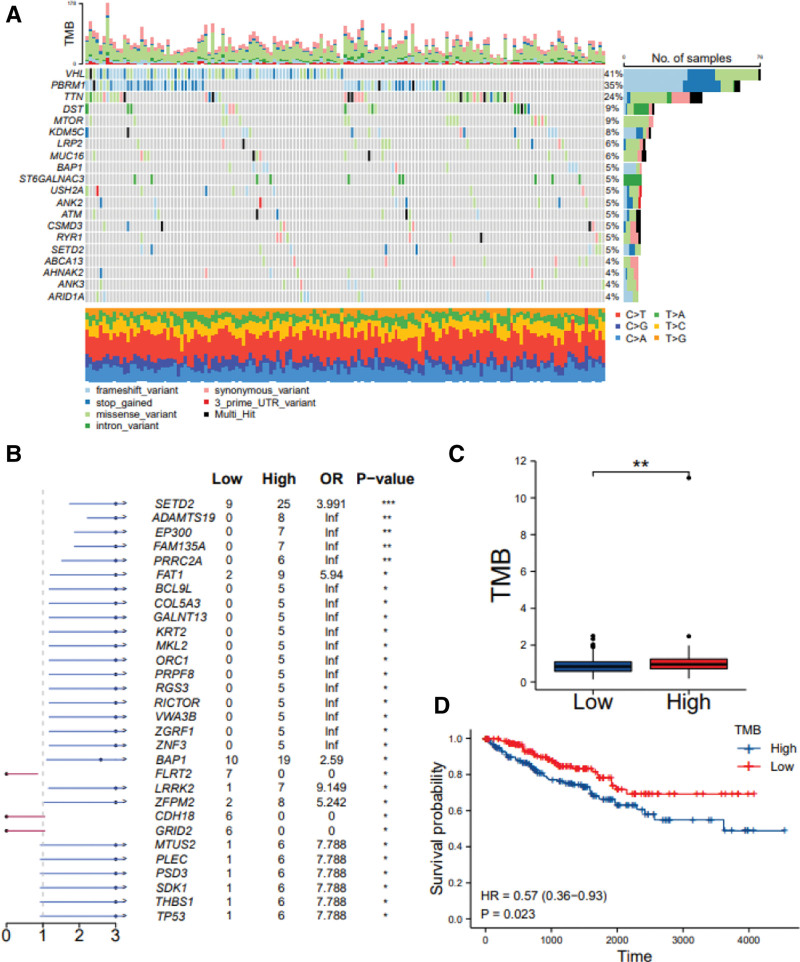
Correlation between somatic mutations and NEK2 expression in ccRCC. (A) Somatic mutations in the NEK2 low expression groups. (B) Comparison of mutations between the high expression group and low expression group of NEK2. (C) Tumor mutational burden (TMB) between the two subgroups based on NEK2 expression. (D) Survival analysis of the various TMB-strategized groups. (*P < 0.05, **P < 0.01, ***P < 0.001).

**Figure 4.**
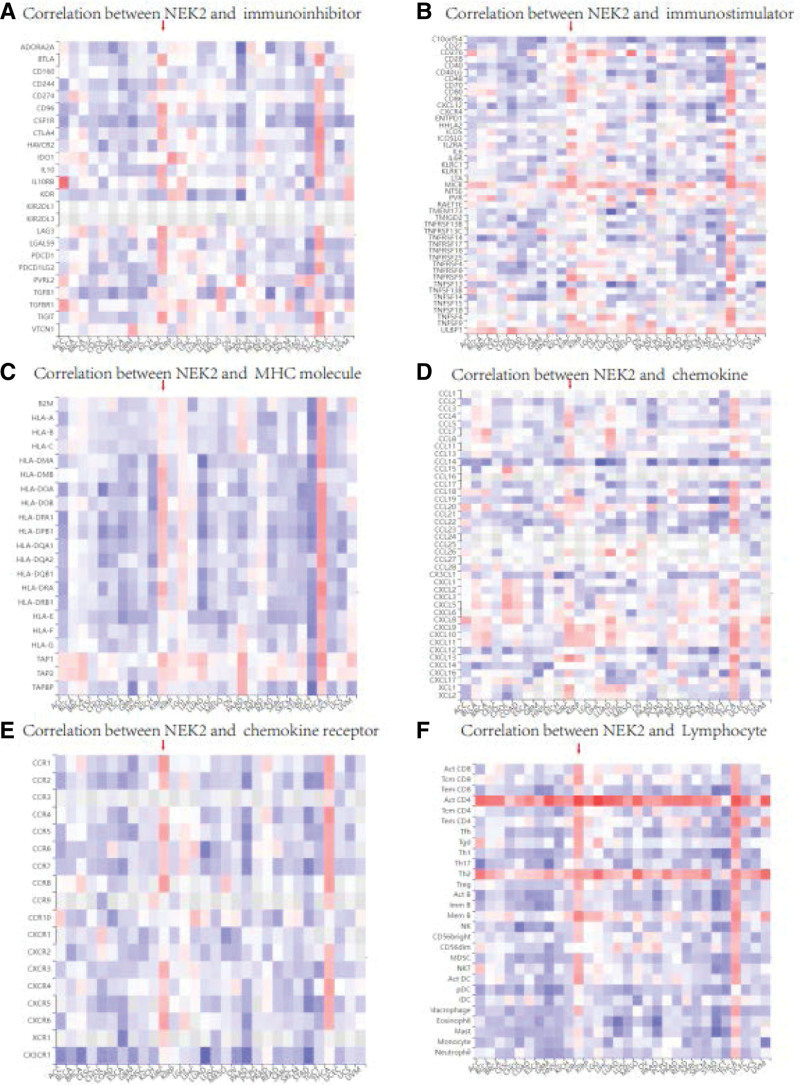
Correlation between somatic mutations and NEK2 expression in ccRCC. (A) Somatic mutations in the NEK2 low expression groups. (B) Comparison of mutations between the high expression group and low expression group of NEK2. (C) Tumor mutational burden (TMB) between the 2 subgroups based on NEK2 expression. (D) Survival analysis of the various TMB-strategized groups (*P < .05, **P < .01, ***P < .001.). ccRCC = clear cell renal cell carcinoma.

**Figure 5.**
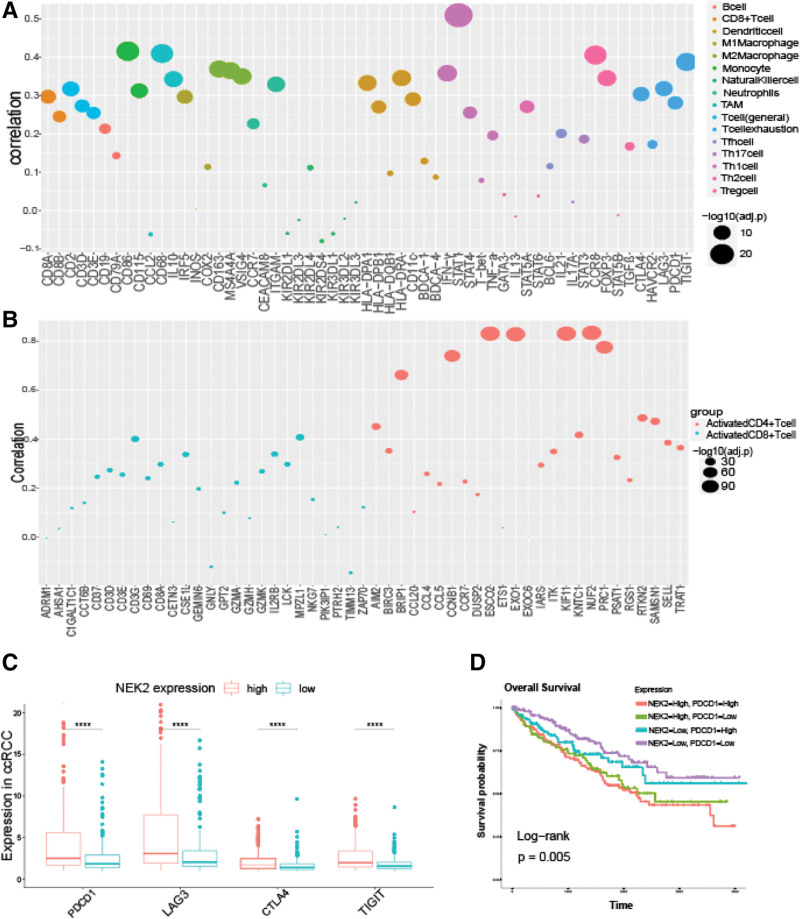
Relevance of NEK2 and immune-related genes (A) Relationship between NEK2 and immune inhibitors. (B) Relationship between NEK2 and immune stimulators. (C) Relationship between NEK2 and MHC molecules. (D) Relationship between NEK2 and chemokines. (E) Relationship between NEK2 and chemokine receptors. (F) Relationship between NEK2 and lymphocytes.

**Figure 6.**
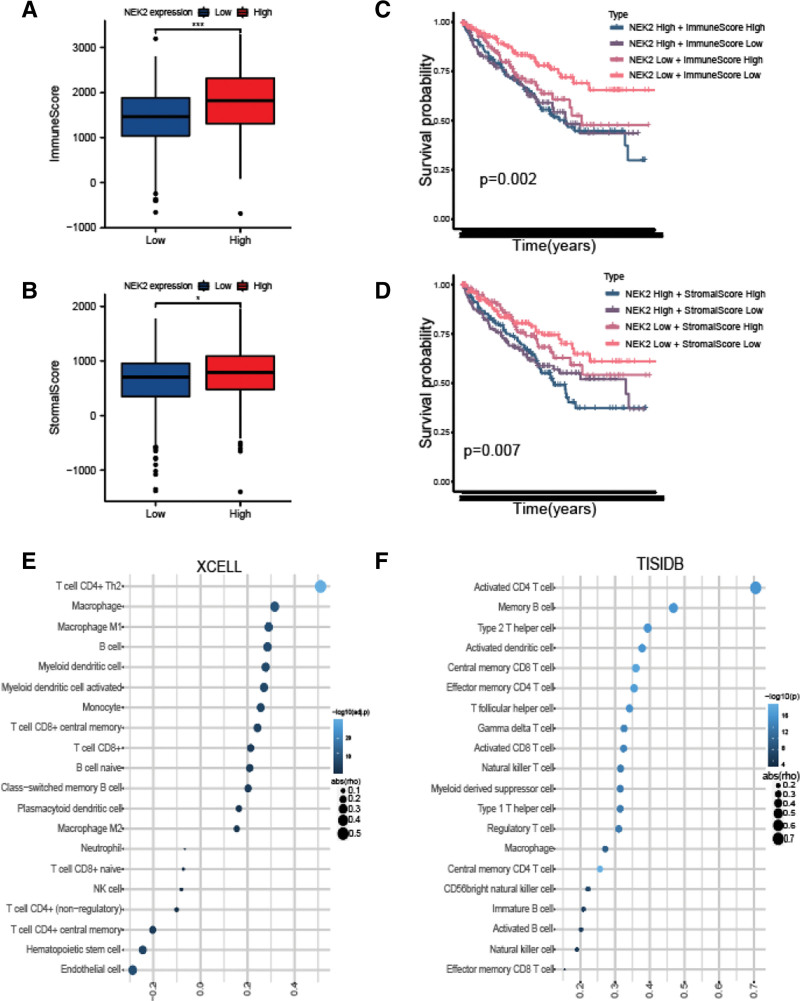
The association between immune cell marker gene sets and NEK2 expression. (A) The bubble plot of the correlations of NEK2 with marker sets of 16 various immune cells. (B) The bubble plot of the correlations of NEK2 with markers of activated CD8+/CD4 + T cells. (C) Box plot of T-cell exhaustion-related immune marker (PDCD1, LAG3, CTLA4 and TIGIT) expression in ccRCC tissues. (D) K-M curves of OS in ccRCC patients based on NEK2 expression and PDCD1 expression. ccRCC = clear cell renal cell carcinoma, OS = overall survival.

### 3.2. Relationship of somatic mutations and tumor mutational burden with NEK2 expression in ccRCC

We acquired the mutation profiles of the KIRC cohort from TCGA database to investigate whether the expression of the NEK2 gene affected the mutations in the ccRCC cohort, taking into account the crucial role of the NEK2 gene in cell proliferation. We drew a mutation map of the 2 different NEK2 expression groups and found that the top 5 mutated genes in the low NEK2 expression group were VHL (41%), PBRM1 (35%), TTN (24%), DST (9%) and MTOR (9%) (Fig. 4A), and those in the other group were VHL (41%), PBRM1 (40%), TTN (27%), SETD2 (17%) and BAP1 (13%) (Supplementary Figure 5, http://links.lww.com/MD/M259). We observed that patients in the NEK2 high group had a significantly higher rate (17%) of SETD2 mutation. Additionally, we discovered that when the mutation rates in the 2 expression groups were compared, the group with higher NEK2 expression had more gene mutations (Fig. 4B). This might be related to the high amplification and DNA replication errors of ccRCC cells with NEK2 overexpression. Subsequently, we further investigated the value of TMB between the 2 subgroups based on NEK2 expression, considering the close association of TMB and immunotherapy effectiveness. Notably, the high NEK2 expression category had a greater TMB and was more likely to benefit from immunotherapy (Fig. 4C). Furthermore, patients with reduced TMB showed a good survival benefit (Fig. 4D).

### 3.3. Correlation between NEK2 expression and various immune markers

To examine the relationship between NEK2 expression and IRGs, we conducted a correlation analysis based on the TISIDB database. Surprisingly, NEK2 was coexpressed with practically all IRGs (Fig. 5A–E). In contrast, in ccRCC samples, KDR (Cor = -0.26, *P* < .0001), PVRL2 (Cor = -0.112, *P* < .01), TNFRSF4 (Cor = -0.227, *P* < .0001) and CX3CL1 (Cor = -0.349, *P* < .0001) all displayed significant negative correlations with NEK2. Furthermore, no connections between NEK2 and the following genes or proteins were found to be significant: ADORA2A, CD160, CD274, IDO1, VTCN1, C10orf54, CD40, CXCL12, ENTPD1, ICOSLG, IL6R, NT5E, PVR, TNFSF25, TNFSF15, HLA-E, HLA-F, HLA-G, CCL-2, CCL-28, CXCL8, CXCL12, CXCL14, CCR10 and CXCR1 (Fig. 5A–E).

To improve our comprehension of the impact of NEK2 on immunological regulation, we made an effort to relate the status of diverse invading immune cells to the relevance of NEK2 expression (Figs. 5F and 6A). We discovered a strong association between the NEK2 gene and markers of T-cell exhaustion and Treg cells, including CCR8, LAG3, PD-1 and TIGIT, after correcting for tumor purity (Supplementary Figure 6A-D, http://links.lww.com/MD/M260). As shown in Figure 6C, the expression of these immune-suppressive substances (PDCD1, LAG3, CTLA4, TIGIT) that are related to T-cell exhaustion was significantly different between the different NEK2 expression subgroups. Furthermore, we discovered that NEK2 overexpression together with markers of T-cell exhaustion, such as PD-1, LAG3, CTLA4 and TIGIT, indicated the worst prognosis in ccRCC (Fig. 6D, Supplementary Figure 7A-C, http://links.lww.com/MD/M261). These data suggested a strong relationship between NEK2 expression levels and T-cell exhaustion, and they also suggested that NEK2 likely plays a significant role in immunological escape in the microenvironment of clear cell RCC. Furthermore, the expression of NEK2 was positively correlated with markers of CD8 + T cells and T cells (general). We examined the relationship between NEK2 expression and indicators of T-cell activation, such as activated CD8 + T cells and CD4 + T cells, to better understand how NEK2 expression and the status of T cells relate to 1 another (Fig. 6B). The plot showed that most markers of T-cell activation were positively related to NEK2 expression in ccRCC, while the immune markers of CD8 + T cells were more strongly correlated. Additionally, there was a significant correlation between NEK2 gene expression in ccRCC and markers of tumor-associated macrophages, M2 macrophages, monocytes, and dendritic cells (Fig. 6A). Using the GEPIA database, we further confirmed the relationship between NEK2 levels and markers of monocytes, tumor-associated macrophages, and M2 macrophages. These data suggested that macrophage polarization in clear cell RCC was influenced by the NEK2 gene.

### 3.4. Characteristics of immune cell infiltration in ccRCC with high NEK2 expression

**Figure 7.**
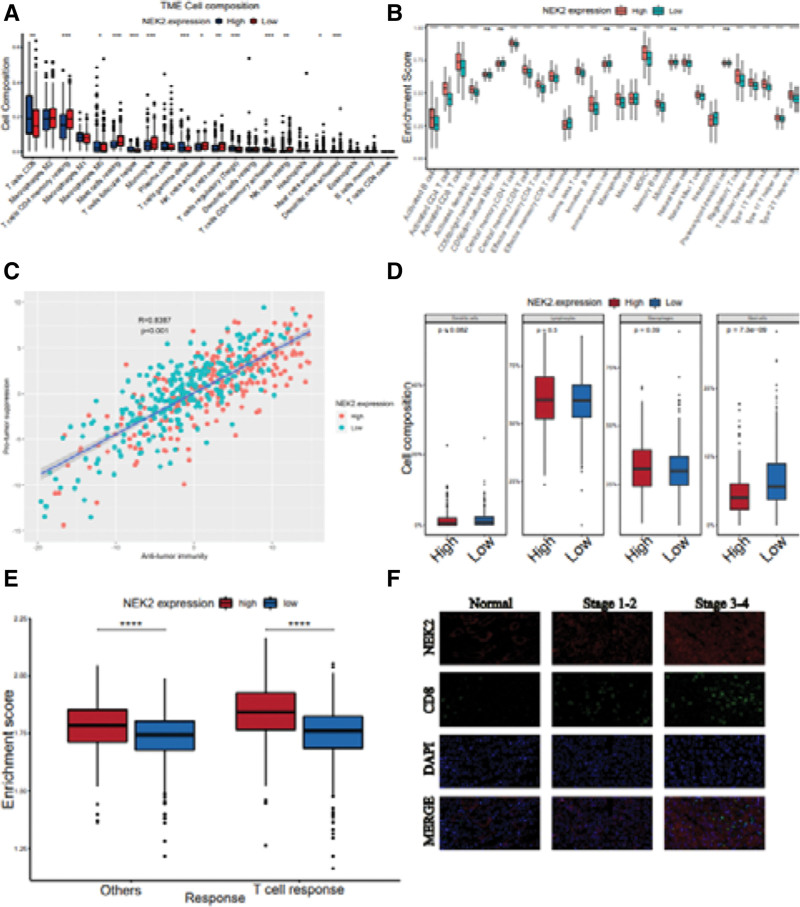
Immune cell infiltration characteristics in ccRCCs expressing NEK2. (A–B) Differences in immune scores and stromal scores between the NEK2-low and NEK2-high expression subgroups (*P < .05, **P < .01, ***P < .001, ****P < .0001). (C–D) KM survival curve of OS based on NEK2 expression levels, immune scores and stromal scores. (E–F) The correlation between NEK2 expression and immune cell infiltrates was analyzed by xCell and TISIDB platforms. ccRCC = clear cell renal cell carcinoma, OS = overall survival.

We examined the relationship between NEK2 expression and markers of different immune cells, but we were still unsure of the characteristics of immune cell infiltration in ccRCC with high NEK2 expression. Therefore, first, the ESTIMATE method was used to examine the connections between NEK2 and stromal or immunological scores. Our findings showed that samples with higher NEK2 expression had significantly higher immunological and stromal scores (Fig. 7A–B). Furthermore, to investigate the correlation between stromal and immunological scores and prognosis, ccRCC samples were separated into high- and low-score subgroups according to the median of the immune and stromal scores. Patients in the subgroup with high immune scores and elevated NEK2 expression had a worse prognosis than those in the subgroup with low immunological scores and low NEK2 expression. Similarly, patients with high stromal scores and strong NEK2 expression had a lower OS than those with low stromal scores and low NEK2 expression (Fig. 7C–D).

Second, using the xCell and TISIDB datasets in ccRCC, we investigated the relationship between NEK2 levels and the invasion of immune cells (Fig. 7E–F). The infiltration of type 2 T helper cells and memory B cells in ccRCC was shown in both plots to be favorably correlated with NEK2 levels. We used the CIBERSORT method to examine the tumor microenvironment (TME) of ccRCC patients with varying NEK2 gene ex-pression to further evaluate the relationship between the makeup of different immune cells and NEK2 gene levels. According to the plot (Fig. 3A), the top 5 immune cell compositions in the TME of patients with ccRCC were CD8 + T cells, M2 macrophages, CD4 + T cells (memory resting), M1 macrophages, and M0 macrophages (Fig. 3A). Additionally, compared to ccRCC patients with low NEK2 expression, individuals with high NEK2 expression exhibited larger proportions of CD8 + T cells and gamma delta T cells (Fig. 3A). The greater infiltration of Treg cells, memory resting CD4 + T cells, and resting mast cells further confirmed the suppressive nature of the TME of ccRCC with high NEK2 expression. Dendritic cells, lymphocytes, macrophages, and mast cells were among the 4 groups created from these 22 different immune cell types, and it was determined how each group differed from the others in terms of NEK2 expression during infiltration (Fig. 3D). The findings demonstrated that the NEK2high group had a comparatively high level of mast cell infiltration, although the proportions of the other 3 categories did not differ significantly between the 2 groups. By applying single-sample gene set enrichment analysis (ssGSEA) in conjunc-tion with mRNA expression profiling of the TCGA-KIRC project, the activities of immune cells were further examined. Based on how immune cells behave in the TME, these immune cells were split into 2 kinds (antitumor immunity and protumor immune-suppressive activities). However, in the ccRCC cohort, the high NEK2 expression group did not differ from the low NEK2 expression group in terms of enhancing tumor activity and antitumor activity (Fig. 3C). Notably, ccRCC with high NEK2 gene expression exacerbated both the T-cell and other immune responses (Fig. 3E). The activity of most immune cells increased in the high NEK2 expression subgroup, including activated or effector memory CD4+, CD8 + T cells and Treg cells (Fig. 3B), which indicated that high NEK2 expression had a strong correlation with immune infiltration in ccRCC. Due to the above findings that NEK2 expression was strongly related to CD8 + T cells, we explored CD8A expression among various tumor stages in the ccRCC cohort. There were significant differences between any 2 tumor stages, and a higher tumor stage was likely to result in higher CD8A expression. Interestingly, through double immunofluorescence staining of NEK2 and CD8 on ccRCC tissue slices, we found mean fluorescence intensity (MFI) of CD8 (green) significantly increased with the growth of tumor stage, along with the increasing MFI of NEK2 (red) (Fig. 3F). It was consistent with bioinformatics results and emphasized the strong correlation of NEK2 with CD8.

### 3.5. Immunotherapeutic responses and drug susceptibility analysis of NEK2 expression

**Figure 8.**
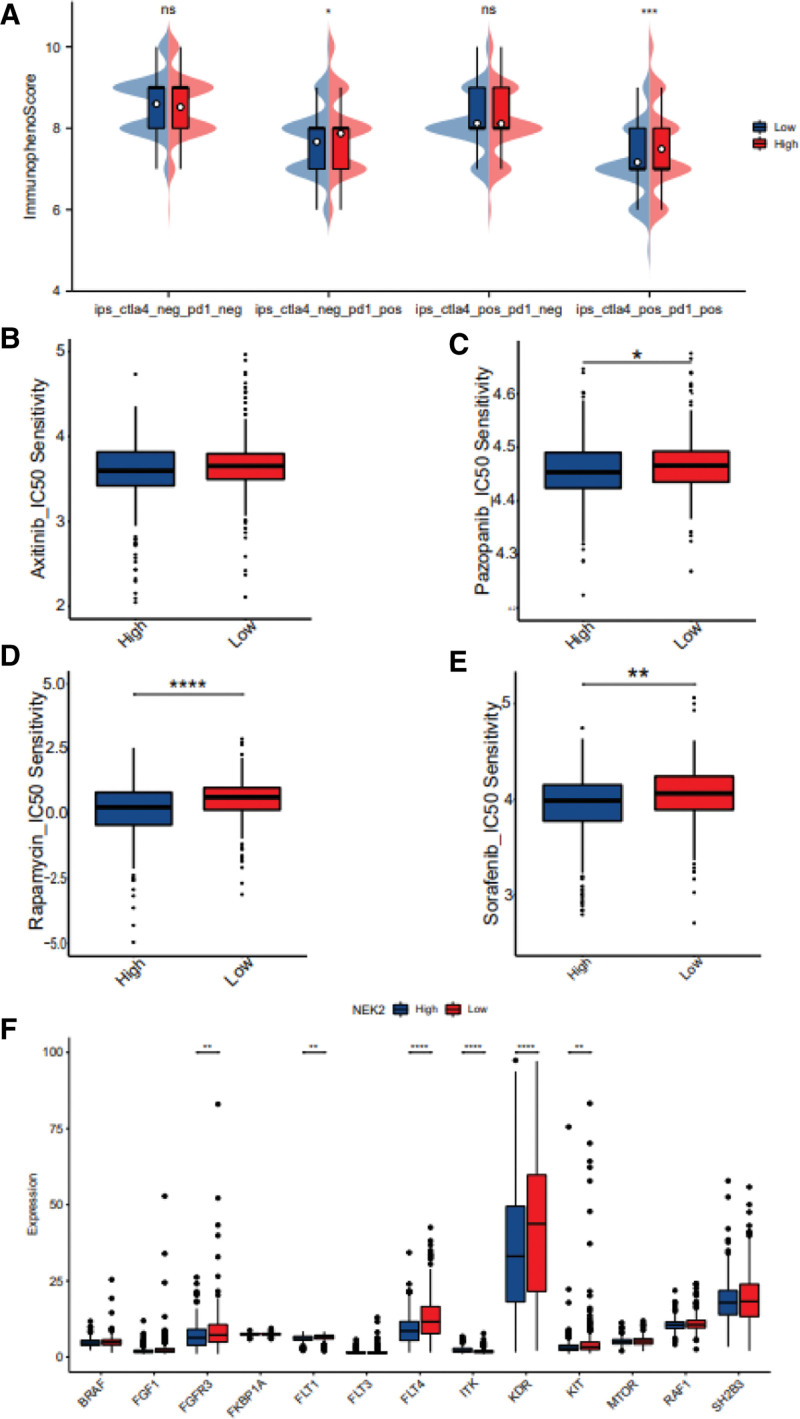
Assessment of therapeutic response through 2 subgroups based on NEK2 expression. (A) Comparing CTLA4 and PD-1-strategized immunophenoscores (IPSs) between the low- and high-NEK2 expression groups. (B-E) Sensitivity analysis for axinib (B), pazopanib (C), rapamycin (D), sorafenib (E) in ccRCC patients with low and high NEK2 expression. (F) Differences between the expression levels of the target genes in the low- and high-NEK2 expression groups following targeted medication therapy. ccRCC = clear cell renal cell carcinoma.

Considering that higher NEK2 expression was associated with poor prognosis, we examined the significance of NEK2 expression to evaluate the impact of immunotherapy utilizing TCIA. The findings showed that the high NEK2 expression group had higher relative probabilities of responding to PD-1 inhibitors alone or in conjunction with CTLA-4 inhibitors than did the low NEK2 expression group (Fig. 8A). However, the sensitivity to CTLA-4 inhibitors alone did not differ between the 2 groupings (Fig. 8A). According to this, patients with high NEK2 expression may respond to CTLA4negative/PD-L1positive and CTLA4positive/PD-L1positive immunotherapy better and have more positive clinical results.

Additionally, we conducted further analysis exploring the relationship between NEK2 expression and drug sensitivity. Using the pRRophetic algorithm, half-maximal inhibitory concentration information was acquired to predict the treatment response. We observed that patients in the high NEK2 expression group were significantly more sensitive to pazopanib, rapamycin, sorafenib, sunitinib and temsirolimus (Fig. 8B–E, Supplementary Figure 8A-B, http://links.lww.com/MD/M262). To assess the effectiveness of targeted drug therapy, we further assessed target gene expression in both groups of ccRCC, which was downloaded from the DrugBank database. The results showed noticeable variations in the expression of most target genes, including FGFR3, FLT1, FLT4, ITK, KDR and KIT, between the 2 groups (Fig. 8F). These results suggested that NEK2 expression may be able to identify ccRCC patients who will respond better to proper treatment

### 3.6. Strongly correlation genes with NEK2 showed proproliferation features in ccRCC cells

**Figure 9.**
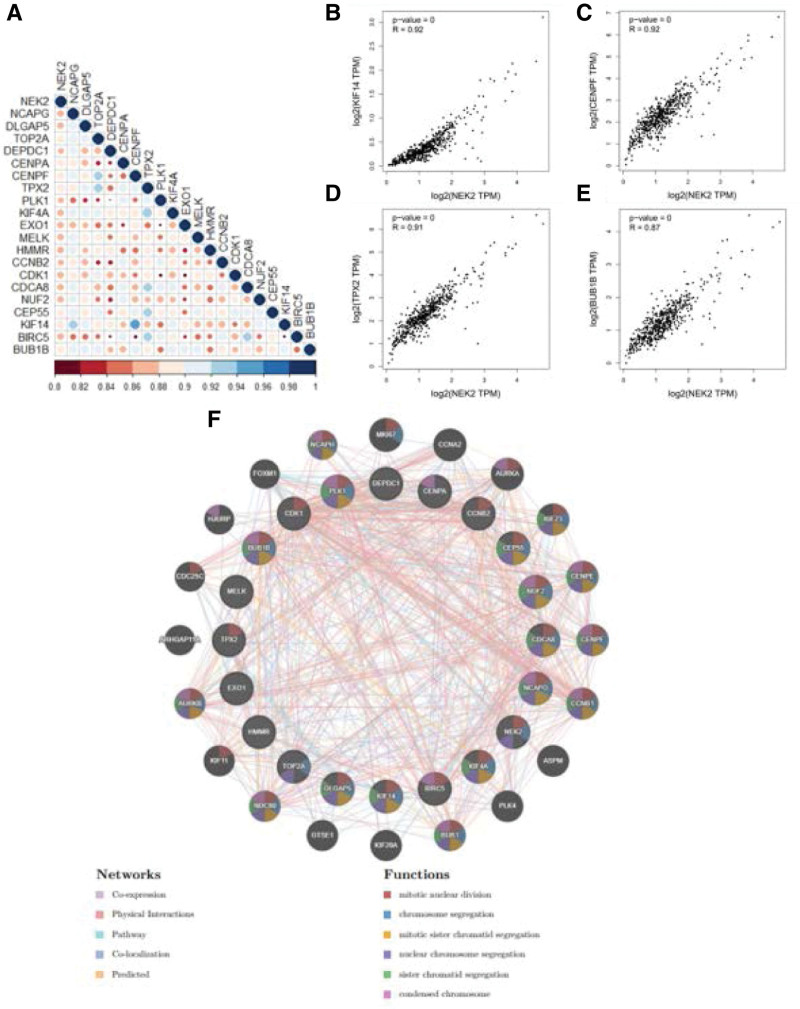
Relationship between NEK2 and coexpressed genes related to the cell cycle in ccRCC. (A) Associations between NEK2 and cell cycle-related coexpressed genes. (B-E) Correlation between NEK2 expression and KIF14, CENPF, TPX2 and BUB1B based on the TIMER database. (F) Visualization of the interaction network of genes strongly associated with NEK2 by GeneMANIA. ccRCC = clear cell renal cell carcinoma.

Then, using coexpression analysis, we identified the genes in the KIRC cohort that were correlated with the expression of NEK2, and we filtered those significantly connected with NEK2 expression (Fig. 9A). We found that the expression of NEK2 was significantly positively correlated with genes linked to the cell cycle, such as KIF14, CENPF, TPX2, and BUB1B (all *R* > 0.85, *P* < .001) (Fig. 9B–E). The functions of coexpressed genes were investigated using GeneMANIA, which also forecasts functionally related genes. These coexpressed genes were found inside the circle, while the predicted genes were outside the circle (Fig. 9F). Their main functions are mitotic nuclear division, chromosomal segregation, and mitotic sister chromatid segregation, all of which are related to NEK2 and may hasten the development of tumors.

**Figure 10.**
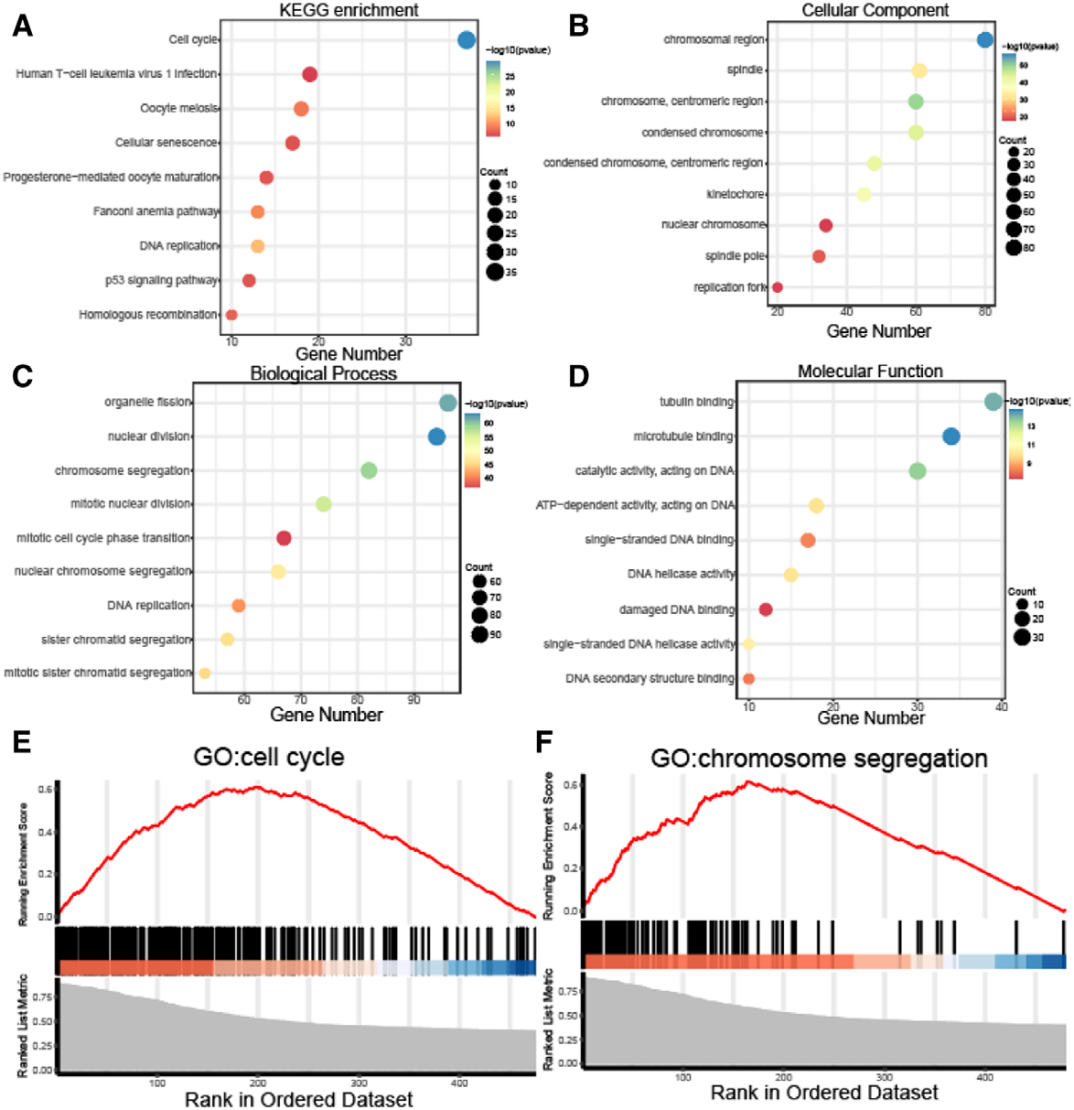
Coexpression genes related to NEK2 showed proliferation-related characteristics. (A–D) KEGG pathway enrichment and GO analysis of 480 strongly correlated genes. (E–F) Gene set enrichment analysis (GSEA) indicated that NEK2 is positively related to the cell cycle and chromosome segregation. GO = gene ontology, KEGG = Kyoto encyclopedia of genes and genomes.

To determine the potential function of the NEK2 gene in ccRCC, we performed coexpression gene analysis of NEK2 in the TCGA-KIRC project. KEGG enrichment analysis was used to analyze the functions of these 480 strongly correlated genes, showing these genes were predominantly enriched in the cell cycle (Fig. 10A). Next GO analysis was used. Organelle fission, nuclear division, chromosomal segregation, and mitotic nuclear division were the biological processes of these coexpressed genes that were most enriched (Fig. 10C), as these processes were connected to cell division and proliferation. These significantly associated genes’ molecular and cellular functions focused mostly on the processes of cell division and proliferation (Fig. 10B and D). Additionally, GSEA was used to identify the signaling pathways that were strongly related to the coexpressed genes. According to the findings (Fig. 10E–F), which were in line with those of KEGG and GO analyses, the expression profiles of the genes correlated with NEK2 were enriched in mitotic sister chromatid segregation, mitotic cell cycle, nuclear division, and cell cycle.

## 4. Discussion

Previous research has shown that the NEK2 gene is crucial for controlling several aspects of the cell cycle, including the stability of microtubules, centrosome replication and division, chromatin condensation, kinetochore adherence, checkpoints in spindle formation, and spindle polar age markers.^[30–34]^ Although the involvement of NEK2 in the morbidity and survival rate of some malignant tumors has been reported,^[35]^ research exploring the roles of NEK2 in the development of clear cell RCC remains mainly unexplored. Thus, this is the first study to evaluate the transcription levels, functions, and connections of NEK2 with prognosis and immune infiltration in patients with clear cell RCC using bioinformatics and integrated data mining of biological databases.

We performed a comprehensive analysis of publicly available data to investigate the potential function of NEK2 in ccRCC and its slight impact on the TME. Transcriptome analysis of samples from the TCGA database and the 2 GEO datasets revealed that NEK2 expression is considerably overexpressed in tumor tissues compared to pa-ra-tumor tissues. This is in line with the earlier investigation.^[36]^ High NEK2 expression also functioned as a poor predictive factor of OS in ccRCC in regard to survival analysis. NEK2 was also linked to prognosis in various malignancies, including pancreatic cancer, hepatocellular cancer, nasopharyngeal cancer, gastric cancer, colorectal cancer, and breast cancer.^[37–44]^ We analyzed NEK2 expression between various tumor stages to better understand the role of NEK2 in the development of ccRCC. We found that patients with advanced tumor stages had higher levels of the NEK2 gene. Additionally, multivariate Cox regression analysis confirmed that elevated NEK2 lev-els were a standalone predictor of poor prognosis. These findings suggested that the NEK2 gene was a useful biomarker for ccRCC prognosis.

To comprehensively understand the characteristics of NEK2 in ccRCC, we identified gene mutations related to NEK2 expression levels. We discovered that SETD2 mutations occurred at a much greater rate (17%) among patients in the NEK2 high group. SETD2 is an H3K36 methyltransferase. It has been reported that SETD2 mutations confer cisplatin resistance in non-small cell lung cancer (NSCLC),^[45]^ enhance epithelial-mesenchymal transition in pancreatic ductal adenocarcinoma,^[46]^ exhibit proinflammatory and proliferation signatures in glioblastoma,^[47]^ and promote widespread DNA hypomethylation in ccRCC.^[48]^ Mutations in the SETD2 gene suppress autophagy and are a frequent phenomenon, causing unfavorable prognosis in ccRCC.^[49,50]^ A recent study showed that most genes correlated with immune activities were upregulated in patients with SETD2 mutant tumors.^[51]^ Notably, it has been reported that SETD2 mutation together with other nonsynonymous mutations drives the response to PD-1 blockade by interacting with CD8 + T-cell infiltration.^[52]^ In short, the subgroup with increased NEK2 expression with high SETD2 mutations may have a worse prognosis than the subgroup with low NEK2 expression with low SETD2 mutations when immunological infiltration is taken into account.

By calculating the Spearman coefficient, we identified some genes that had a positive correlation with NEK2, such as KIF14, CENPF, TPX2 and BUB1B. Interestingly, these genes have been reported to be involved in the cell cycle and immune infiltration, resulting in poor prognosis in tumors.^[53–57]^ According to our study findings from KEGG enrichment, GO analysis, and GSEA, tumor cells with high levels of NEK2 expression were very active during the cell cycle, chromosome segregation, and nuclear division processes. It also clarified why patients with tumors that overexpressed the NEK2 gene and were associated with a higher percentage of somatic mutations had worse clinical outcomes than patients whose tumors expressed lower levels of NEK2 and why the overexpression NEK2 phenotype in the ccRCC cohort is significantly correlated with some clinical features, such as T stages and pathological stages. In summary, the above results suggest that NEK2 may be implicated in tumor immunity in ccRCC.

Our work demonstrated that the NEK2 gene was associated with the immunological infiltration of different immunocytes in ccRCC, which had not been previously investigated. According to the findings, type 2 T helper cell and memory B-cell infiltration into ccRCC was substantially correlated with NEK2 levels. Interestingly, it was similar to previous studies.^[44]^ Our further research showed that the transcriptional level of the NEK2 gene was closely related to markers of Treg cells, T-cell exhaustion, CD8 + T cells and T cells (general) in ccRCC. This suggests that ccRCC tumors overexpressing the NEK2 gene may be a hot tumor for 1 day because our findings indicated a strong link between NEK2 expression and the majority of markers of activated T cells in ccRCC, specifically CD8+/CD4 + T cells. Additionally, there were favorable connections between the expression of NEK2 levels and the levels of Treg cell and T-cell exhaustion markers such CCR8, FOXP3, TIGIT, and PDCD1. These results are in line with recent studies,^[58]^ revealing that these markers provide a potential target for tumor intervention. CCR8 induced Ca2 + flux via its ligand CCL1 and potentiated the in vivo proliferation and suppressive activities of Treg cells.^[59]^ By inhibiting the immune system from keeping track of abnormal cells, FOPX3 produced a setting for the proliferation of malignant cells.^[60]^ PD-L1 can induce an inhibitory signal in T cells and inhibit tumor-related T-cell responses.^[61]^ Through a variety of mechanisms, such as preventing NK-cell degranulation, cytokine production, and NK-cell-mediated cytotoxicity of tumor cells expressing CD155, TIGIT effectively inhibited both innate and adaptive immunity.^[62]^ An earlier study showed that Treg cells given by FOXP3 may inhibit antitumor immune responses by suppressing self-antigen responses, which would produce an immunosuppressive environment.^[63]^ The progression of ccRCC was significantly associated with higher Tregs.^[64]^ Due to its favorable correlation with PD-L1, NEK2 has recently been recognized as a prognostic factor in immunologically “hot” pancreatic cancer.^[65]^ Interestingly, NEK2 overexpression showed increased infiltration of CD8 + T cells but decreased infiltration of CD4 + memory T cells. It is worth noting that both previous^[66]^ and recent^[67]^ data show that increased CD8 + T-cell infiltration is associated with a worse outcome in ccRCC, which is different in other tumor types. Increased concentrations of PD-1 and polyclonal CD8 + T cells with poor cytotoxic capacity may be the underlying processes.^[68]^ Meanwhile, in our study, high NEK2 expression indicated increased infiltration of Tregs. Notably, earlier research has shown that Tregs are overrepresented in ccRCC and that higher densities of these cells are associated with a worse prognosis.^[69,70]^ It was implied that patients with NEK2 overexpression would have poor prognoses and some degree of immunosuppression.

Targeted therapy and immune checkpoint inhibitors (ICIs) are crucial first-line therapies for the treatment of KIRC.^[29]^ Nevertheless, there still exist challenges to decide which treatment is best for individuals. Both CTLA4 and PD-1 are essential ICIs. In our study, we discovered a statistically significant difference between the 2 NEK2 expression groups in patients receiving effective CTLA4 and PD-L1 treatments. Patients with various NEK2 expression levels could also respond differently to pazopanib, rapamycin, sorafenib, sunitinib and temsirolimus therapies. Target genes for these medications, such as FGFR3, FLT1, FLT4, ITK, KDR and KIT, showed substantial changes between the 2 groups, which was consistent with the differing sensitivity to these drugs. These results suggested that the NEK2 gene may be a notable marker for identifying ccRCC patients who will respond better to proper immunotherapy and targeted therapy.

This study is subject to several limitations. First, our investigation into NEK2 function in ccRCC was based on data from the GEO, TCGA, and online databases, some of which were validated by our validation cohort. We did not, however, perform in vivo and in vitro experiments to affirm NEK2 role in the biological processes of the cell cycle and DNA replication or its connection to immune cell infiltration in the TME. Future mechanistic experiments are needed to clarify the underlying mechanism underlying the trend observed. Second, we lacked a clinical cohort that contained a large sample size and long follow-up to test and verify the conclusions obtained from the online database. Finally, additional in-depth investigation is required to confirm its clinical importance in directing immunotherapy.

## 5. Conclusions

In summary, this study reveals that NEK2 is an underlying immune-related prognostic biomarker and may help in predicting outcomes, distinguishing immune and molecular characteristics and guiding proper therapies in ccRCC patients.

## Acknowledgments

The authors would like to thank TCGA projects for providing high-quality clinical data on clear cell renal carcinoma. Also, we are grateful for participation and cooperation from renal cell carcinoma patients

## Author contributions

**Data curation:** Peng Tang, Gangfu zheng, Congcong Xu, Jiaqi Du, Liqian Hu

**Formal analysis:** Peng Tang, Congcong Xu, Nengfeng Yu, Liqian Hu

**Funding acquisition:** Peng Tang, Liqian Hu, Zhan Zhou, Yichun Zheng

**Methodology:** Peng Tang, Congcong Xu, Nengfeng Yu

**Resources:** Peng Tang, Gangfu zheng, Zhan Zhou, Yichun Zheng

**Software:** Peng Tang, Jiaqi Du

**Validation:** Peng Tang

**Writing – original draft:** Peng Tang

**Writing – review & editing:** Peng Tang

## Supplementary Material
