# Supplementary material for: Function of NEK2 in clear cell renal cell carcinoma and its effect on the tumor microenvironment
Source: Medicine (Baltimore). 2024 May 17;103(20):e37939. doi: 10.1097/MD.0000000000037939 (PMC11098263; doi:10.1097/MD.0000000000037939)
Supplement: Supplementary file 1 [file medi-103-e37939-s001.docx]

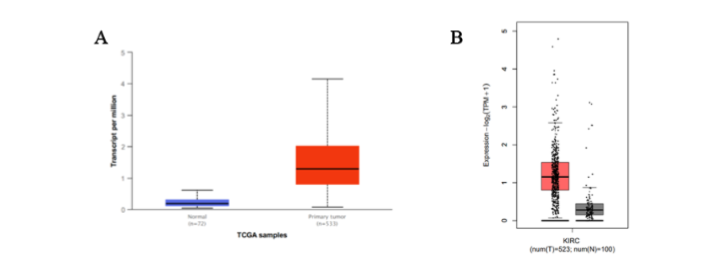


Supplementary Figure 1

Box plot of NEK2 expressions in tumor and non-tumor tissues from the UALCAN (A) and GEPIA (B) database.
