# Supplementary figures and images for: Function of NEK2 in clear cell renal cell carcinoma and its effect on the tumor microenvironment
Source: Medicine (Baltimore). 2024 May 17;103(20):e37939. doi: 10.1097/MD.0000000000037939 (PMC11098263; doi:10.1097/MD.0000000000037939)

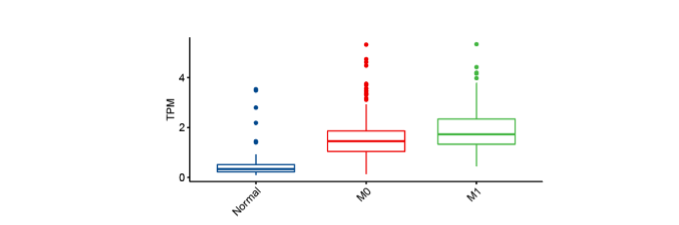


Supplementary Figure 2

Correlation between NEK2 expression level and tumor metastasis stage.

Supplement: Supplementary file 2 [file medi-103-e37939-s002.docx]

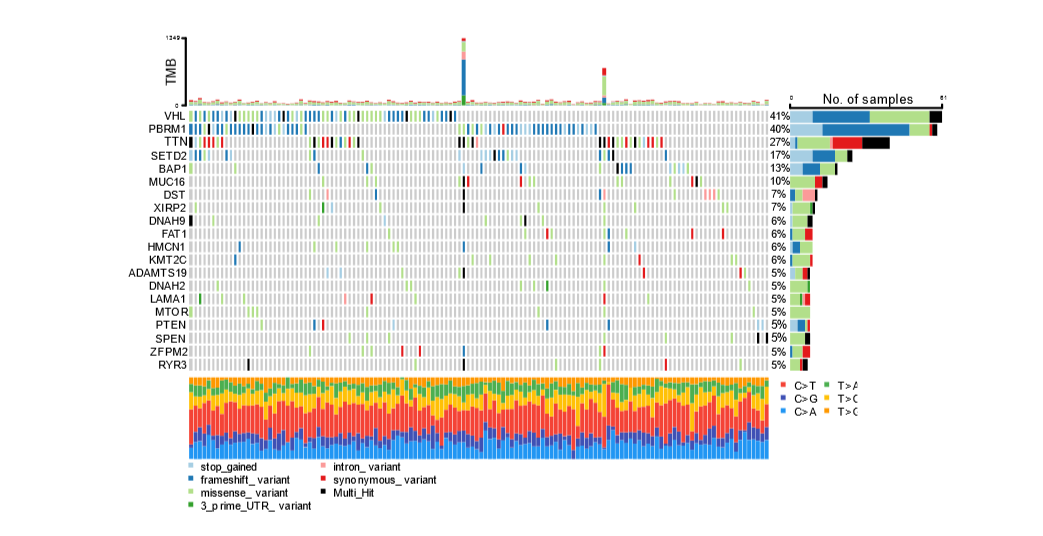


Supplementary Figure 5

Somatic mutations in the NEK2 high expression groups.

Supplement: Supplementary file 5 [file medi-103-e37939-s005.docx]
