# Supplementary material for: Function of NEK2 in clear cell renal cell carcinoma and its effect on the tumor microenvironment
Source: Medicine (Baltimore). 2024 May 17;103(20):e37939. doi: 10.1097/MD.0000000000037939 (PMC11098263; doi:10.1097/MD.0000000000037939)
Supplement: Supplementary file 3 [file medi-103-e37939-s003.docx]

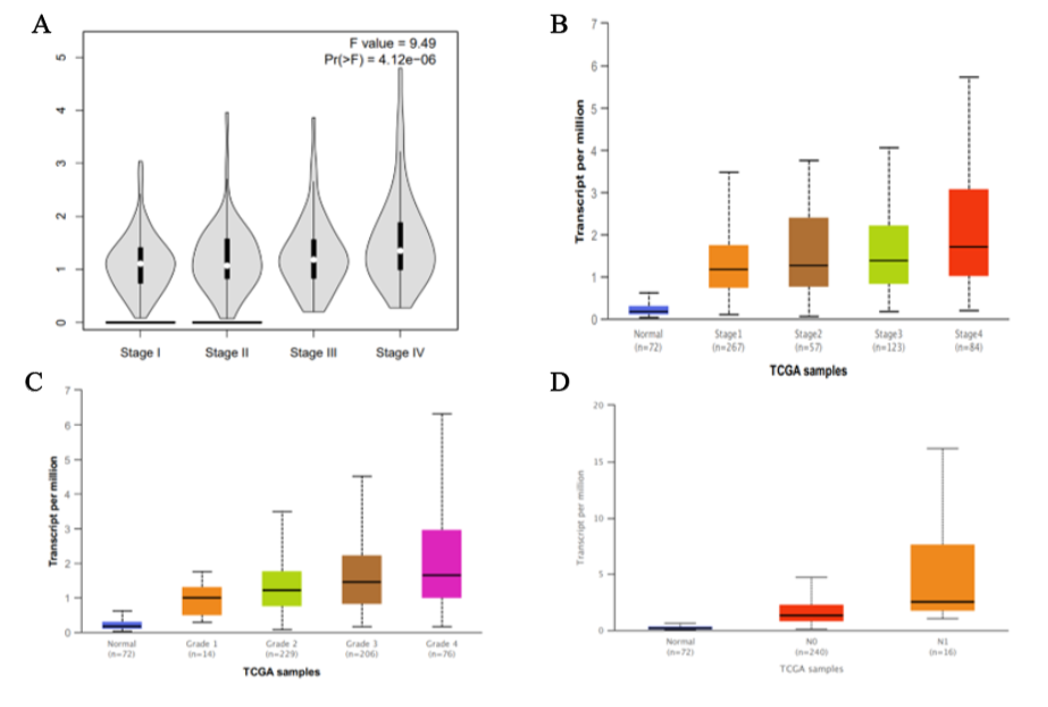


Supplementary Figure 3

NEK2 expression levels along with clinicopathological characteristics in ccRCC. (A-D) Correlation between NEK2 expression level and clinicopathological parameters (cancer stage, cancer grade and lymph node stage) of ccRCC through the GEPIA and UALCAN database.
