# Supplementary material for: Function of NEK2 in clear cell renal cell carcinoma and its effect on the tumor microenvironment
Source: Medicine (Baltimore). 2024 May 17;103(20):e37939. doi: 10.1097/MD.0000000000037939 (PMC11098263; doi:10.1097/MD.0000000000037939)
Supplement: Supplementary file 6 [file medi-103-e37939-s006.docx]

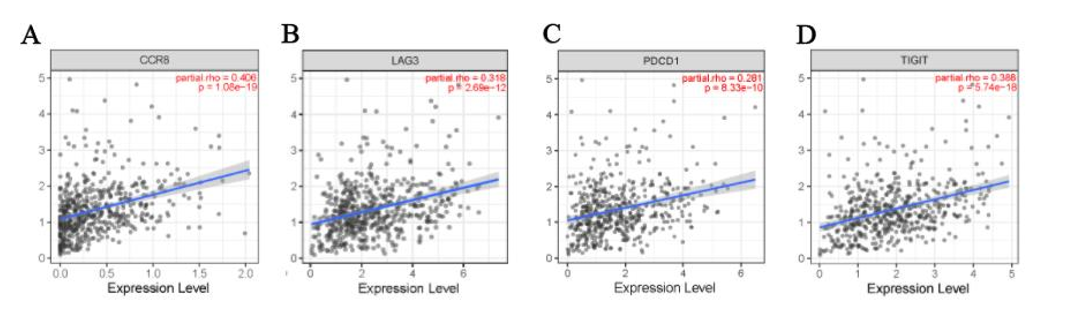


Supplementary Figure 6

Correlations between the expression of EOGT and immune-suppressive substances (CCR8 (A), LAG3 (B), PDCD1 (C), TIGIT (D)) that are related to T-cell exhaustion.
