# Supplementary material for: Function of NEK2 in clear cell renal cell carcinoma and its effect on the tumor microenvironment
Source: Medicine (Baltimore). 2024 May 17;103(20):e37939. doi: 10.1097/MD.0000000000037939 (PMC11098263; doi:10.1097/MD.0000000000037939)
Supplement: Supplementary file 7 [file medi-103-e37939-s007.docx]

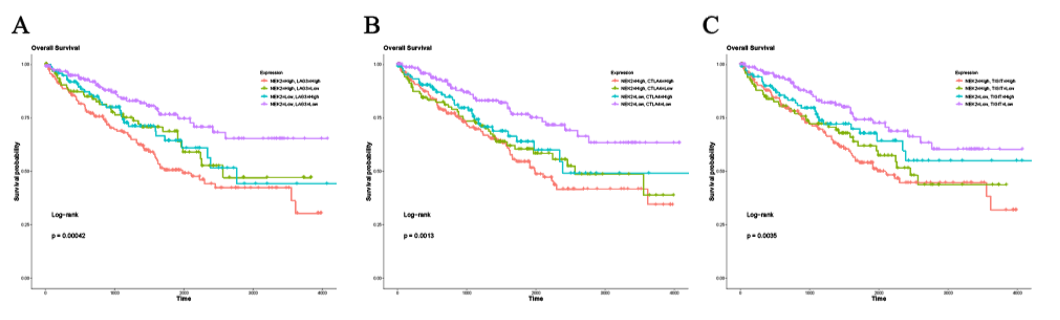


Supplementary Figure 7

K-M curves of OS in ccRCC patients based on NEK2 expression and LAG3 (A), CTLA4 (B) and TIGIT (C) expression.
