# Supplementary material for: Function of NEK2 in clear cell renal cell carcinoma and its effect on the tumor microenvironment
Source: Medicine (Baltimore). 2024 May 17;103(20):e37939. doi: 10.1097/MD.0000000000037939 (PMC11098263; doi:10.1097/MD.0000000000037939)
Supplement: Supplementary file 8 [file medi-103-e37939-s008.docx]

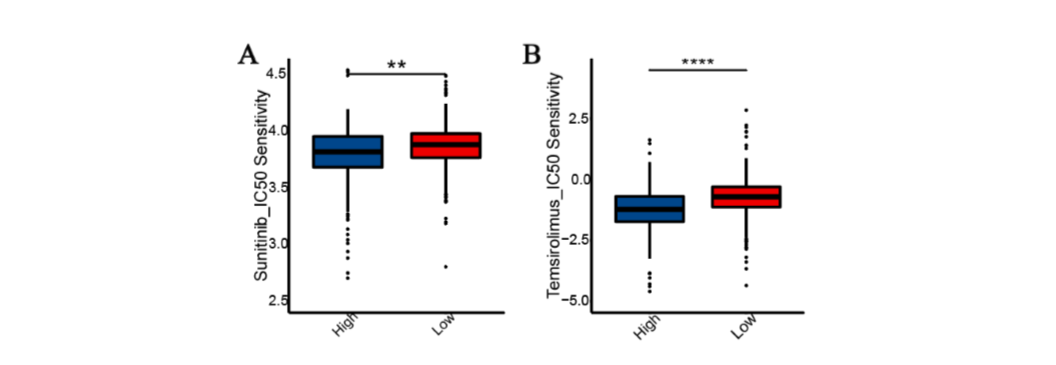


Supplementary Figure 8

Sensitivity analysis for sunitinib (A) and temsirolimus (B) in ccRCC patients with low and high NEK2 expression.
